# Factors Driving Attrition from Neoadjuvant Therapy to Pancreatectomy in Localized Pancreatic Cancer

**DOI:** 10.1245/s10434-025-18664-0

**Published:** 2025-11-15

**Authors:** Erin M. Dickey, Lucy Min, Annika Dhawan, Mary Martos, Dido Franceschi, Alan S. Livingstone, Peter Hosein, Gretel Terrero, Jashodeep Datta, Nipun Merchant, Caitlin Hester

**Affiliations:** 1 Division of Surgical Oncology, Dewitt Daughtry Department of Surgery, University of Miami Miller School of Medicine, Miami, FL USA; 2 Sylvester Comprehensive Cancer Center, Miami, FL USA; 3 Department of Medicine, University of Miami Miller School of Medicine, Miami, FL USA

**Keywords:** Neoadjuvant therapy, Localized pancreatic cancer, Rates of attrition, Rates of pancreatectomy after neoadjuvant therapy, Rates of attrition according to radiographic stage

## Abstract

**Background:**

Neoadjuvant therapy (NAT) has become the cornerstone in the treatment of localized pancreatic cancer (LPC), with the goal of downstaging tumors, treating occult metastases, and selecting patients with favorable biology for surgery. Although the benefits of NAT have been well described, real-world data on attrition rates and barriers against proceeding to pancreatectomy are limited.

**Methods:**

This study analyzed LPC patients treated with at least one cycle NAT at a high-volume center (2011–2022). The patients were stratified by radiographic stage (resectable [R], borderline resectable [BR], locally advanced [LA]), or pancreatectomy status. Reasons for attrition during NAT and survival outcomes were evaluated.

**Results:**

Among 427 LPC patients who received NAT (R, 57 [13%]; BR, 133 [31%]; and LA, 237 [56%]), 182 (43%) underwent pancreatectomy. The overall attrition rate of 57% during NAT was driven by disease progression (21%), persistent inoperability (22%), physiologic decline (11%), loss to follow-up evaluation (2%), and surgery refusal (1%). Attrition rates correlated with stage (R [23%], BR [44%], LA [73%]; *p* < 0.01). The median overall survival (OS) was significantly longer for the resected patients (30 vs 16 months; *p* < 0.01) and inversely correlated with stage (R [30 months], BR [21 months], and LA [18 months]; *p* < 0.01). Overall survival was similar across stages among the resected patients after NAT (R [31 months], BR [27 months], and LA [34 months]; *p* = 0.66).

**Conclusions:**

Stage-stratified attrition during NAT remains a significant issue even for patients with R disease. The similar OS across stages for resected patients highlights NAT’s role in selecting those most likely to benefit from surgery and underscores the need for strategies to reduce attrition.

**Supplementary Information:**

The online version contains supplementary material available at 10.1245/s10434-025-18664-0.

Pancreatic adenocarcinoma (PDAC) is projected to become the second leading cause of cancer-related deaths by 2030.^1^ The majority of patients present with locally advanced or metastatic disease, and survival remains poor even among those receiving curative-intent therapy as per National Comprehensive Cancer Network (NCCN) guidelines.^2^ The ideal sequence of surgery and chemotherapy for patients with localized PDAC is debated. However, a neoadjuvant therapy (NAT) approach is increasingly adopted based on the rationale of treating occult metastatic disease and downstaging locally advanced tumors to resectability. It also serves as a strategy for selecting patients with favorable biology who will benefit from resection.^3–5^

Although the benefits of NAT have been well described, attrition rates and reasons for failure to proceed to pancreatectomy after NAT remain underreported in clinical practice. Existing data are primarily from neoadjuvant clinical trials that enroll highly selected patients, typically those with resectable (R)^6–16^ or borderline resectable (BR)^4,5,13–23^ tumors, and often exclude locally advanced (LA)^24–26^ disease. Several meta-analyses have pooled these trials to address this limited diversity, reporting pancreatectomy rates of 65% to 74% for R tumors and approximately 33% for BR and LA tumors,^27–31^ highlighting the variability in rates of pancreatectomy after NAT.

The real-world data outlining rates of attrition and treatment trajectories for all patients with localized pancreatic cancer (LPC) undergoing NAT are sparse. Providers often counsel patients that NAT serves as a biologic selection tool for those likely to benefit from pancreatectomy, yet stage-specific attrition rates are rarely discussed.

This study evaluated the intent-to-treat attrition rates and reasons for failure to proceed to pancreatectomy among all LPC patients undergoing NAT at a high-volume center. We also examined how attrition rates differ by anatomic stage.

## Methods

### Patient Selection and Characteristics

The clinical records of patients with LPC at a single high-volume tertiary referral center between January 2011 and January 2022 were retrospectively reviewed. We first assessed trends in NAT and upfront resection (UR) to understand trends in curative-intent strategies over time. Only patients who underwent at least one cycle of NAT were included in the attrition analysis.

Radiographic stage was defined according to AHPBA/SSO/SSAT guidelines as resectable (R), borderline resectable (BR), or locally advanced (LA).^32^ Radiographic images were reviewed by board-certified surgical oncologists, who confirmed the radiographic stage. We analyzed the following variables: patient demographics (age, sex, race, ethnicity, American Society of Anesthesiologists [ASA] class, Eastern Cooperative Oncology Group [ECOG] score, and body mass index [BMI]), clinical and tumor characteristics (stage at presentation, tumor location, tumor size, and baseline CA 19-9), NAT details (regimen, duration, cycles of chemotherapy, chemotherapy switches, side effects, radiation therapy), surgical data (resection, aborted surgery, perioperative outcomes), and pathologic details (College of American Pathologists [CAP] tumor regression grade,^33^ margin status–pancreatic neck, unicate, and common bile duct margin, lymph node metrics, lymphovascular invasion, perineural invasion). The chemotherapy regimens included modified FOLFIRINOX (mFOLFIRINOX), gemcitabine/abraxane (GA), or other. Pathologic response was graded using the CAP tumor regression grading system.^33^

### Institutional NAT Protocol

Individualized patient treatment decisions were made by a multidisciplinary review board consisting of surgical oncology, medical oncology, radiation oncology, pathology, diagnostic and interventional radiology, and interventional gastroenterology. Use of radiation in the neoadjuvant setting was decided based on response to neoadjuvant chemotherapy by the review board. At our institution, it is not routine practice to include neoadjuvant radiation in the treatment plan unless there is a persistent tumor-arterial interface after neoadjuvant chemotherapy.

### Outcomes

The patients were stratified by whether they underwent pancreatectomy after NAT or did not. The primary outcome was the rate of attrition, defined as failure to undergo pancreatectomy. The reasons for attrition included progression (local progression or development of metastatic disease during NAT), physiologic decompensation (functional decline during NAT precluding surgery), persistent inoperability (due to vascular involvement despite NAT), loss to follow-up evaluation (patients who initiated treatment at our institution but did not return), declined surgery (those who were offered surgery but declined). Classification was based on documentation by board-certified medical oncologists and surgical oncologists. We also stratified patients by anatomic stage to determine how stage at diagnosis affects attrition rates and whether reasons for attrition differ by stage. The secondary outcome was overall survival (OS), defined as time from diagnosis to death or last follow-up visit.

### Statistical Analysis

Characteristics were compared using chi-square or Fisher’s exact test for categorical variables and Student’s *t*, ANOVA, or Mann-Whitney *U* test for continuous variables. Survival analysis used the Kaplan-Meier method with the log-rank test. Multivariable logistic regression identified factors associated with attrition, reported as odds ratios (ORs) with 95% confidence intervals (CIs).

Statistical significance was determined by *p* values lower than 0.05. Statistical analyses were performed via SPSS version 28 (IBM, New York, NY, USA).

## Results

### Trends in Approach: NAT Versus Upfront Resection

**Fig. 1:**
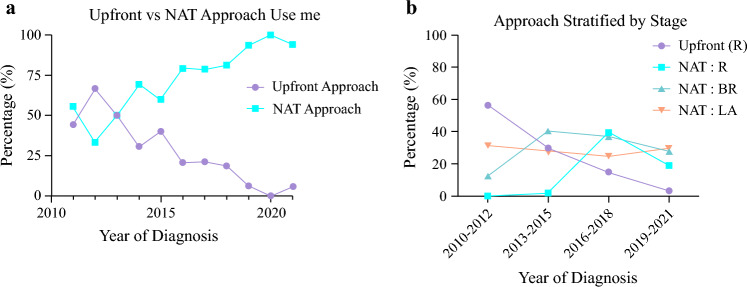
Yearly trends of patients undergoing pancreatectomy stratified by approach: upfront resection versus (**a**) neoadjuvant therapy (NAT) and (**b**) disease stage

Between 2011 and 2022, PDAC was diagnosed for 476 patients, of whom 49 underwent upfront surgery and 427 underwent NAT. The rate of NAT significantly increased during the study period (Fig. 1a; *p* < 0.01). The proportion of patients with R or BR tumors treated with NAT increased significantly over time, whereas NAT use for LA tumors remained consistent during all time periods (Fig. 1b). Henceforth, this study focused on the 427 patients who underwent NAT.

### Patients with Localized PDAC Who Underwent NAT

*NAT Patient Clinical Demographics.*<PUB1> The study cohort included 427 LPC patients treated with NAT. The median age was 66.3 years (interquartile range [IQR], 59–73 years). Half (50%) of the patients were female. In terms of ethnicity, 87% were white and 42% were Hispanic. Most of the patients had an American Anesthesiology Association (ASA) score of at least 3 (56%) and an ECOG score lower than 2 (85%). The tumors were predominantly in the pancreatic head (64%) with a median size of 3.3 cm (IQR, 2.7–4.2 cm). The baseline median CA 19-9 level was 267 U/mL (IQR, 58–766 U/mL). By stage, 57 (13%) tumors were R, 133 (31%) tumors were BR, and 237 (56%) tumors were LA. The NAT regimens included mFOLFIRINOX (60%), GA (28%), or another 5-FU or gemcitabine-based regimen (12%). The median duration of NAT was 4.2 months (IQR, 2.5–5.7 months). Of the 427 patients, 93 (22%) switched regimens. Of the 129 patients (30%) who underwent neoadjuvant radiation therapy, 104 received stereotactic body radiation or image-guided radiation (81%), and 18 received conventional radiation (14%) (Table 1).

**Table 1:** Clinicodemographic and neoadjuvant therapy characteristics of localized pancreatic cancer patients who underwent pancreatectomy after neoadjuvant therapy and those who did not

Variables	Total	Pancreatectomy	Attrition	*p*-value
	*n *(%)	*n* (%)	*n* (%)	
Median age: years (IQR)	66.3 (59.0–73.0)	65.0 (58.0–73.0)	67.2 (60.0–74.0)	0.067
Sex				0.41
Female	213 (49.9)	95 (52.2)	118 (48.2)	
Race				0.33
White	373 (87.4)	164 (90.1)	209 (85.3)	
Black	35 (8.2)	12 (6.6)	23 (9.4)	
Other	19 (4.4)	6 (3.3)	13 (5.3)	
Ethnicity				0.192
Hispanic	181 (42.4)	85 (46.7)	96 (39.2)	
ASA ≥3^a^	161 (56.3)	83 (62.4)	78 (51.0)	0.052
ECOG ≥2^s^	52 (14.9)	16 (11.1)	36 (17.3)	0.107
Median BMI: kg/m^2^ (IQR)	25 (22.5–28.7)	24.9 (22.5–28.6)	25.0 (22.4–28.9)	0.831
Presentation				**0.014**
Incidental	29 (6.8)	16 (8.8)	13 (5.3)	
Jaundice	172 (40.3)	80 (44.0)	92 (37.6)	
Abdominal pain	177 (41.5)	60 (33.0)	117 (47.8)	
Other	49 (11.4)	26 (14.3)	23 (9.4)	
Tumor location				**<0.001**
Head	274 (64.2)	136 (74.7)	138 (56.3)	
Neck	32 (7.4)	9 (4.9)	23 (9.4)	
Body	63 (14.8)	22 (12.1)	41 (16.7)	
Tail	12 (2.8)	6 (3.3)	6 (2.4)	
Multiple	46 (10.8)	9 (4.9)	37 (15.1)	
Median radiographic tumor size: cm (IQR)^c^	3.3 (2.7–4.2)	3 (2.5–3.8)	3.5 (2.9–4.5)	**<0.001**
Radiographic stage				**<0.001**
Resectable	57 (13.3)	44 (24.2)	13 (5.3)	
Borderline resectable	133 (31.1)	74 (40.7)	59 (24.1)	
Locally advanced	237 (55.5)	64 (35.2)	173 (70.6)	
Median CA 19-9 at dx: U/mL (IQR)^d^	266.7 (57.7–766.1)	199.8 (59.2–570.1)	260 (56.5–943.5)	0.232
Initial neoadjuvant regimens				**0.003**
FOLFIRINOX	254 (59.6)	121 (66.9)	133 (54.3)	
Gemcitabine/nab-paclitaxel	122 (28.6)	49 (27.1)	73 (29.8)	
Other	50 (11.7)	11 (6.1)	39 (15.9)	
Median neoadjuvant chemotherapy duration: months (IQR)	4.2 (2.5–5.7)	3.9 (2.6–5.4)	4.4 (2.3–6.3)	0.702
Neoadjuvant cycles	7 (4–10)	7 (5–9)	7 (4–10)	0.462
Neoadjuvant chemotherapy regimen switches	93 (21.8)	26 (14.3)	67 (27.3)	**0.001**
Side effects				**0.003**
None	169 (39.6)	83 (45.6)	86 (35.1)	
Dose reduction	173 (40.5)	76 (41.8)	97 (39.6)	
Treatment Stopped	85 (19.9)	23 (12.6)	62 (25.3)	
Biliary complications	15 (3.5)	10 (5.5)	5 (2.0)	0.055
Neoadjuvant radiation	129 (30.2)	39 (21.4)	90 (36.7)	**<0.001**

Significant *p*-values (*p* < 0.05) are denoted in bold

IQR, interquartile range; ASA, American Society of Anesthesiologists; ECOG, Eastern Cooperative Oncology Group; BMI, body mass index

^a^*n* = 286, 33% missing

^b^*n* = 352, 18% missing

^c^*n* = 408, 5% missing

^d^*n* = 382, 11% missing

### Rates of Pancreatectomy and Attrition

**Fig. 2:**
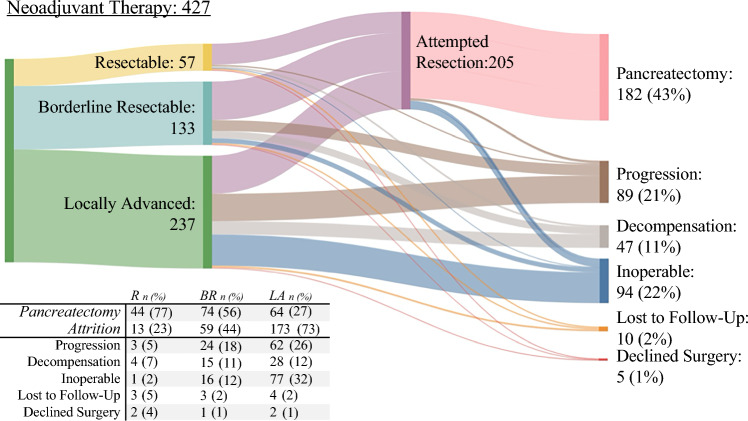
Sankey diagram displaying number of patients who underwent pancreatectomy after neoadjuvant therapy and reasons for failure to proceed to pancreatectomy. Rates are noted in the table below. Sankey diagram created via SankeyMATIC.com

Of the 427 patients who received NAT, 205 (48%) were deemed operable, and 182 (43%) who went to the operating room for underwent a pancreatectomy. Surgery was aborted or palliative for 23 (5%) of the 205 patients due to occult metastases, with 18 (78%) of the 23 patients deemed inoperable. Attrition during NAT was 57% because of progression (21%), persistent inoperability (22%), physiologic decline (11%), loss to follow-up evaluation (2%), or declined surgery (1%) (Fig. 2).

### Comparison of Patients Who Underwent Pancreatectomy Versus No Pancreatectomy

The patients who underwent pancreatectomy had smaller tumors (median, 3.0 cm [IQR, 2.5–3.8 cm] vs 3.5 cm [IQR, 2.9–4.5 cm]; *p* < 0.001) and lower anatomic staging (R [24% vs 5%], BR [41% vs 24%], LA [35% vs 71%]; *p* < 0.001) than those who did not undergo pancreatectomy. There were no differences in age, sex, race, ethnicity, ASA class, or ECOG status.

**Fig. 3:**
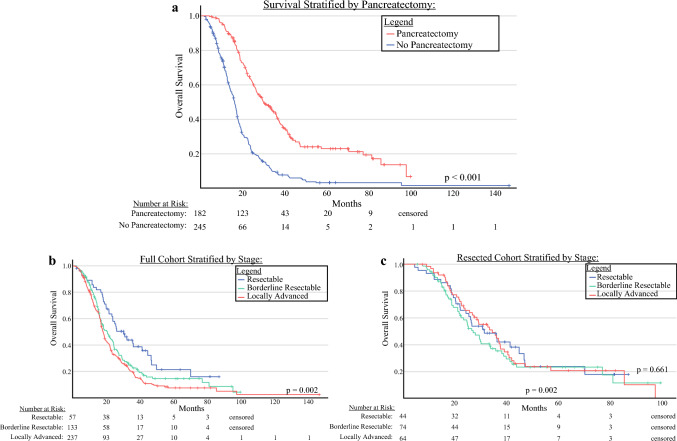
Kaplan Meier analyses depicting overall survival (OS) between (**a**) the patients who underwent a pancreatectomy after neoadjuvant therapy (NAT) (29.6 months; 95% CI, 24.2–35.0 months) and those who did not (16 months; 95% CI, 14.5–17.4 months), (**b**) the full cohort stratified by stage (R: 30.4 months [95% CI: 22.5–38.2 months], BR: 20.8 months [95% CI, 16.7–25.0 months], LA: 18.2 months [95% CI, 16.7–19.7 months]; *p* = 0.002), and (**c**) the resected cohort stratified by stage (R: 31.1 months [95% CI, 20.3–41.8 months], BR: 26.8 months [95% CI, 21.7–31.9 months], LA: 32.2 months [95% CI, 26.1–42.2 months]; *p* = 0.661). R, resectable; BR, borderline resectable; LA, locally advanced

Duration of NAT was similar between the groups (3.9 vs 4.4 months; *p* = 0.700). The unresected patients had higher rates of chemotherapy switch (27% vs 14%; *p* = 0.001) and neoadjuvant radiation therapy (37% vs 21%; *p* < 0.001). During NAT, the cohorts did not differ in terms of biliary complications such as cholecystitis, cholangitis, or stent manipulation (5.5% vs 2.0%; *p* = 0.05; Table 1). The patients who underwent pancreatectomy had longer median OS (29.6 months [95% CI, 24.2–35.0 months] vs 16.0 months [95% CI, 14.5–17.4 months; *p* < 0.001; Fig. 3a).

### Odds of Attrition

**Fig. 4:**
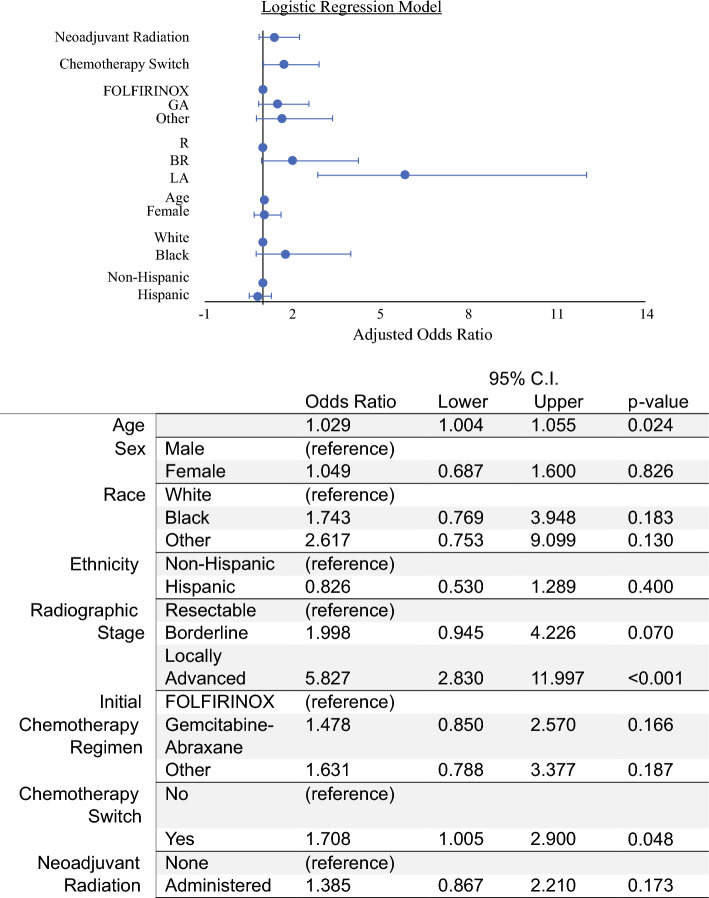
Multivariate logistic regression model for factors associated with odds of attrition

Multivariable analysis showed no association between attrition and sex, race, or ethnicity. The odds of attrition increased with increasing age (OR, 1.029; 95% CI, 1.01–1.1) and more advanced radiographic stage (OR, 5.8 for LA [95% CI, 2.8–12.0] vs R), and among those who had neoadjuvant chemotherapy switch during treatment (OR, 1.7; 95% CI, 1.01–2.9) (Fig. 4).

### Clinical Demographics by Anatomic Stage

Age, sex, race, ethnicity, ASA, and ECOG status were similar across stages (R, BR, LA). Higher stage was associated with larger tumors (*p* < 0.001). Chemotherapy regimens (0.051) and duration (0.142) were similar across stages, whereas regimen-switching (9% vs 17% vs 27%; *p* = 0.003) and neoadjuvant radiation therapy (4% vs 27% vs 38%) were more frequent for the patients with higher stages (Table 2). The median OS decreased with stage (R, 30.4 months [95% CI, 22.5–38.2 months], BR, 20.8 months [95% CI, 16.7–25 months], LA, 18.2 months [95% CI, 16.7–19.7 months]; *p* = 0.002; Fig. 3b).

**Table 2:** Clinicodemographic, neoadjuvant therapy, and pathologic characteristics of patients with resectable, borderline resectable, and locally advanced disease who received neoadjuvant therapy

Variables	Resectable	Borderline	Locally advanced	*p* Value
	(*n* = *57*)	(*n* = *133*)	(*n* = *237*)	
	*n* (%)	*n* (%)	*n* (%)	
Median a*ge*: years* (IQR)*	68.0 (59.0–75.5)	67.0 (59.0–73.0)	66.0 (60.0–73.0)	0.76
Sex				0.815
Female	29 (50.9)	69 (51.9)	115 (48.5)	
Race				0.473
White	53 (93)	188 (88.7)	202 (85.2)	
Black	2 (3.5)	11 (8.3)	22 (9.3)	
Other	2 (3.5)	4 (3.0)	13 (5.5)	
Ethnicity				0.467
Hispanic	23 (40.4)	64 (48.1)	94 (39.7)	
ASA ≥3^a^	28 (58.3)	56 (61.5)	77 (52.4)	0.365
ECOG ≥2^b^	7 (14.9)	18 (16.4)	27 (13.8)	0.834
Median BMI: kg/m^2^ (IQR)	24 (22.7–28.2)	25.2 (22.9–29.0)	25.6 (22.3–28.4)	0.486
Presentation				**<0.001**
Incidental	12 (21.1)	6 (4.5)	11 (4.6)	
Jaundice	29 (50.0)	67 (50.4)	76 (32.1)	
Abdominal pain	11 (19.3)	45 (33.8)	121 (51.1)	
Tumor location				**<0.001**
Head	46 (80.7)	102 (76.7)	126 (53.2)	
Neck	1 (1.8)	7 (5.3)	24 (10.1)	
Body	7 (12.3)	11 (8.3)	45 (19.0)	
Tail	2 (5.3)	3 (2.3)	6 (2.5)	
Spanning	0 (0.0)	10 (7.5)	36 (15.2)	
Median radiographic tumor size: cm (IQR)^c^	2.5 (2.1–3.5)	3.1 (2.7–3.9)	3.6 (3–4.6.0)	**<0.001**
Median CA 19-9 at dx: U/mL (IQR)^*d*^	157.5 (34.2–353.4)	287 (69.7–943.0)	249 (62.0–768.9)	0.05
Initial neoadjuvant regimens				0.051
FOLFIRINOX	34 (59.6)	81 (60.9)	139 (58.9)	
Gemcitabine/ Nab-paclitaxel	22 (38.6)	38 (28.6)	62 (26.3)	
Other	1 (1.8)	14 (10.5)	35 (14.8)	
Median neoadjuvant chemotherapy duration: months (IQR)	3.3 (2.3–5.4)	4.6 (2.6–5.8)	4.4 (2.6–6.1)	0.142
Neoadjuvant cycles	6 (4–8)	7.1 (5–8)	7 (4–10)	0.126
Neoadjuvant Chemotherapy regimen switches	5 (8.8)	23 (17.3)	65 (27.4)	**0.003**
Side effects				0.342
None	19 (33.3)	60 (45.1)	90 (38.0)	
Dose reduction	26 (45.6)	53 (39.8)	94 (39.7)	
Treatment stopped	12 (21.1)	20 (15)	53 (22.4)	
Neoadjuvant radiation	2 (3.5)	36 (27.1)	91 (38.4)	**<0.001**

Significant *p*-values (*p* < 0.05) are denoted in bold

IQR, interquartile range; ASA, American Society of Anesthesiologists; ECOG, Eastern Cooperative Oncology Group; BMI, body mass index

^a^*n* = 286, 33% missing

^b^*n* = 352, 18% missing

^c^*n* =408, 5% missing

^d^*n* = 382, 11% missing

### Rates of Pancreatectomy and Attrition by Anatomic Stage

Pancreatectomy rates declined with stage (R, 77%; BR, 56%; LA, 27%; *p* < 0.001), whereas rates of attrition increased with stage (R. 23%; BR, 44%; LA, 73%; *p* < 0.001). The main reasons for attrition were progression (R, 5%; BR, 18%; LA, 26%), persistent inoperability (R, 2%; BR, 12%; LA, 32%), and physiologic decline (R, 7%; BR, 11%; LA, 12%) (Fig. 2).

### Procedure and Surgical Pathology by Anatomic Stage

The most common procedure performed was pancreatoduodenectomy (76%; R, 82%; BR, 69%; LA, 69%; *p* = 0.265). The LA patients had higher rates of arterial resection (13%) than the BR (2%) and R (0%) patients (*p* = 0.002). The arteries resected were the celiac artery (55.6%), the superior mesenteric artery (22.2%), and the common hepatic artery (22.2%). The groups had similar R0 rates (R, 91%; BR, 85%; LA, 83%; *p* = 0.561), node positivity (*p* = 0.754), CAP score (*p* = 0.084), perineural invasion (*p* = 0.268), and lymphovascular invasion (*p* = 0.097), as well as similar 30-day mortality (R, 5%; BR, 6%; LA, 2%; *p* = 0.477) (Table 3).

**Table 3:** Operative and pathologic outcomes for patients who underwent neoadjuvant therapy (NAT) followed by pancreatectomy stratified the anatomic stage

Variables	Total	Resectable	Borderline	Locally advanced	*p Value*
	(*n* = *182*)	(*n* = *44*)	(*n* = *74*)	(*n* = *64*)	
	*n* (%)	*n* (%)	*n* (%)	*n* (%)	
Operation					0.265
Pancreatoduodenectomy	138 (75.8)	36 (81.8)	58 (78.4)	44 (68.8)	
Distal pancreatectomy	31 (17.0)	8 (18.2)	9 (12.2)	14 (21.9)	
Subtotal pancreatectomy	8 (4.4)	0 (0.0)	3 (4.1)	5 (7.8)	
Total pancreatectomy	4 (2.2)	0 (0.0)	3 (4.1)	1 (1.6)	
Central pancreatectomy	1 (0.5)	1 (1.4)	1 (1.4)	0 (0.0)	
Arterial resection	9 (4.9)	0 (0.0)	1 (1.4)	8 (12.5)	**0.002**
SMA	2 (1.1)	0 (0.0)	0 (0.0)	2 (3.1)	**0.028**
Celiac artery	5 (2.7)	0 (0.0)	0 (0.0)	5 (7.8)	
Hepatic artery	2 (1.1)	0 (0.0)	1 (1.4)	1 (1.6)	
Surgical margin^a^					0.561
R0	155 (85.6)	40 (90.9)	62 (84.9)	53 (82.8)	
R1	25 (13.8)	4 (9.1)	11 (15.1)	10 (15.6)	
R2	1 (0.6)	0 (0.0)	0 (0.0)	1 (1.6)	
Lymph node status					0.754
Positive^a^	86 (47.5)	23 (52.3)	33 (45.2)	30 (46.9)	
Median no. of positive^a^ lymph nodes (IQR)^a^	0 (0.0–2.0)	1 (0.0–2.8)	0 (0.0–2.0)	0 (0.0–2.0)	0.836
Median pathologic size: cm (IQR)^b^	2.4 (1.5–3.2)	2.5 (1.5–3.2)	2.4 (1.4–3.2)	2.3 (1.6–3.3)	0.881
Pathologic differentiation^b^					0.660
G0	7 (3.9)	1 (2.3)	4 (5.5)	2 (3.1)	
G1	4 (2.2)	2 (4.7)	2 (2.7)	0 (0.0)	
G2	104 (57.8)	21 (48.8)	43 (58.9)	40 (62.5)	
G3	61 (33.9)	18 (41.9)	23 (31.5)	20 (31.3)	
Not graded	4 (2.2)	1 (2.3)	1 (1.4)	2 (3.1)	
CAP score^c^					0.084
0	8 (4.7)	2 (4.9)	4 (5.7)	2 (3.3)	
1	25 (14.5)	4 (9.8)	9 (12.9)	12 (19.7)	
2	81 (47.1)	15 (36.6)	36 (51.4)	30 (49.2)	
3	51 (29.7)	18 (43.9)	21 (30.0)	12 (19.7)	
Not scored	7 (4.1)	2 (4.9)	0 (0.0)	5 (8.2)	
Perineural invasion^b^	116 (64.4)	31 (70.5)	44 (60.3)	41 (65.1)	0.268
Lymphovascular invasion^a^	72 (39.8)	19 (43.2)	33 (45.2)	20 (31.3)	0.097
30-Day mortality	7 (3.9)	2 (4.5)	4 (5.5)	1 (1.6)	0.477

Significant *p*-values (*p* < 0.05) are denoted in bold

SMA, superior mesenteric artery; IQR, interquartile range; CAP, College of American Pathologists

^a^*n* = 181, 0.5% missing

^b^*n* = 180, 1.1% missing

^c^*n* = 172, 5.5% missing

### Survival by Stage in Pancreatectomy Patients

Although fewer patients with higher stages proceeded to pancreatectomy, those who underwent resection had similar median OS (R, 31.1 months [95% CI, 20.3–41.8 months]; BR, 26.8 months [95% CI, 21.7–31.9 months]; LA, 32.2 months [95% CI, 26.1–42.2 months]; *p* = 0.661; Fig. 3b).

### Variables Associated with Survival

In a Cox regression analysis, advanced radiographic stage (HR, 1.8; 95% CI, 1.1–3.1 for BR [*p* = 0.025]; HR, 1.8; 95% CI, 1.1–3.0 for LA [*p* = 0.020] compared with R) and attrition status (HR, 3.0; 95% CI, 2.1–4.3 [*p* < 0.001] compared with pancreatectomy) were significantly associated with increased mortality (Table S1).<ST1>

## Discussion

This study has provided one of the first comprehensive real-world evaluations of attrition rates and reasons for failure to proceed to pancreatectomy among all comers who have LPC treated with NAT. Our findings reflect evolving practice patterns, with a clear shift toward NAT as the preferred initial strategy for potentially curative-intent treatment across all stages of LPC. Importantly, this analysis included all patients regardless of their performance status, comorbidities, or stage, offering insight into outcomes beyond the constraints of the highly selective inclusion criteria of clinical trials.

We observed an overall attrition rate during NAT of 57% in our cohort, predominantly driven by disease progression and persistent inoperability. As anticipated, attrition correlated strongly with radiographic stage and inversely with OS. A key and reassuring finding was that OS is comparable across all LPC stages among patients who undergo NAT followed by pancreatectomy, reinforcing the critical role of surgery for well-selected patients, even those with initial LA disease. This supports the concept that NAT functions as an effective “biologic filter,” identifying patients most likely to benefit from resection.

Our attrition rates for R (23%) and BR (44%) cases align with those reported in landmark clinical trials, such as PREOPANC, SWOG S1505, and ESPAC-5F.^10,11,16,23^ Notably, our patients were less selected, suggesting that these rates are achievable outside clinical trial settings. In contrast, LA PDAC has long been considered inoperable due to concerns about surgical futility and morbidity.^34–36^ However, accumulating evidence, including our data, indicates that a subset of LA PDAC patients can undergo successful resection after prolonged NAT, with outcomes similar to those for R or BR disease. Most clinical trials of LA PDAC patients focus on chemotherapy or chemoradiotherapy without surgery.^37–39^ Only the NEOLAP-AIO-APK-0113 trial assessed the role of induction chemotherapy in surgical outcomes.^26^ Our 27% resection rate for LA PDAC is comparable with NEOLAP’s 32% rate and exceeds the rate seen in other reported series (e.g., 3% in Gulhati et al.^35^ and 16% in a published meta-analyses by Damm et al.^40^).^26^ Our data not only demonstrated that LA PDAC patients had pathologic and survival outcomes similar to those of R and BR patients, but also demonstrated the value of NAT in identifying those patients who will benefit from aggressive surgical interventions.

The 27% conversion rate from LA to resectable disease not only highlights the downstaging potential of NAT, but also underscores the need for more effective systemic therapies capable of achieving higher rates of conversion to resection. Future strategies should include novel agents such as immunotherapy as well as KRAS inhibitors and biomarker-driven approaches to better identify early responders.

Our lower rate of attrition (11%) attributable to physiologic decline during NAT compared with 15% to 46% in other reports likely reflects our institution’s flexibility in delivering NAT regimens, and our ability to deliver more individualized treatment plans contributes meaningfully to patient resilience and response through therapy.^10,11,41,42^ This highlights the importance of ongoing multidisciplinary evaluation during NAT.

Despite the advantages of NAT, critics of this approach caution about the risk of missing the “optimal surgical window.”^43,44^ Our data suggest otherwise, showing that only 5% of patients with initially resectable tumors progressed during NAT, and that only 7% experienced decompensation precluding their ability to undergo a pancreatectomy. These findings demonstrate that NAT effectively identifies patients with biologically aggressive disease and those with limited physiologic reserve, sparing them from non-beneficial surgery. Our data also emphasize the importance of setting realistic patient expectations and providing precise counseling regarding the benefits of a NAT approach and the reality of attrition.

Our study’s strengths included its large, unselected cohort and detailed documentation of attrition causes. However, several limitations inherent to the study design should be noted. First, its retrospective nature introduced potential variability in staging and restaging assessments. Second, it likely had inherent biases that appear to be associated with attrition, including use of radiation. In reality, radiation is selected for patients who have more advanced tumors with a persistent tumor-arterial interface, thus those more likely to not proceed with resection. Third, robust, objective data regarding quantifiable social support metrics were not systemically collected in this database. We acknowledge that the lack of social determinants of health analysis was a critical omission, but efforts are ongoing to collect these data to specifically address the impact of social factors on attrition. Additionally, our database did not thoroughly explore variables related to prehabilitation efforts other than baseline functional status (ECOG/ASA), which was not a major driver of attrition. Finally, our database did not capture the grade of chemotherapy adverse events.

In conclusion, this real-world study highlighted a 57% attrition rate among LPC patients undergoing NAT, predominately among those with LA disease. Our findings underscored the critical role of NAT as a powerful selection tool to identify patients most likely to benefit from pancreatectomy and highlighted the ongoing need for effective neoadjuvant strategies to provide more treatment opportunities to patients with localized disease. Finally, no difference in OS was seen for the patients receiving all curative-intent treatment regardless of stage, further supporting NAT for selection of patients most likely to benefit from resection.

## Supplementary Information

Below is the link to the electronic supplementary material.

Supplementary file1 (DOCX 19 kb)
