# Supplementary material for: Factors Driving Attrition from Neoadjuvant Therapy to Pancreatectomy in Localized Pancreatic Cancer
Source: Ann Surg Oncol. 2025 Nov 15;33(3):2642–52. doi: 10.1245/s10434-025-18664-0 (PMC12901226; doi:10.1245/s10434-025-18664-0)
Supplement: Supplementary file 1 — Supplementary file1 (DOCX 19 kb) [file 10434_2025_18664_MOESM1_ESM.docx]

**Supplemental Table 1:** Cox regression model for factors associated with mortality.

| **Variable** |  | **Hazard Ratio** | **95% C. I.**  **Lower Upper** | | **p-value** |
| --- | --- | --- | --- | --- | --- |
| Increasing Age |  | 1.007 | 0.988 | 1.026 | 0.481 |
| Race | Caucasian | (reference) |  |  |  |
|  | Black | 0.734 | 0.409 | 1.317 | 0.299 |
|  | Other | 1.299 | 0.596 | 2.829 | 0.510 |
| ASA | <3 | (reference) |  |  |  |
|  | ≥3 | 1.221 | 0.881 | 1.693 | 0.230 |
| ECOG | <2 | (reference) |  |  |  |
|  | ≥2 | 1.196 | 0.763 | 1.875 | 0.435 |
| Sex | Male | (reference) |  |  |  |
|  | Female | 0.698 | 0.509 | 0.956 | **0.025** |
| Hispanic | Non-Hispanic | (reference) |  |  |  |
|  | Hispanic | 0.833 | 0.596 | 1.165 | 0.285 |
|  | Unreported | 1.455 | 0.150 | 14.080 | 0.746 |
| Increasing BMI |  | 0.969 | 0.938 | 1.001 | 0.061 |
| Presentation | Incidental | (reference) |  |  |  |
|  | Jaundice | 0.651 | 0.361 | 1.176 | 0.155 |
|  | Pancreatitis | 0.470 | 0.182 | 1.211 | 0.118 |
|  | Weight Loss | 1.462 | 0.408 | 5.242 | 0.560 |
|  | Pain | 0.620 | 0.341 | 1.126 | 0.116 |
|  | Other | 0.549 | 0.211 | 1.433 | 0.221 |
|  | Diabetes | 0.472 | 0.058 | 3.847 | 0.483 |
| Tumor Location | Head | (reference) |  |  |  |
|  | Neck | 1.009 | 0.557 | 1.828 | 0.976 |
|  | Body | 1.030 | 0.646 | 1.642 | 0.902 |
|  | Tail | 0.229 | 0.055 | 0.957 | **0.043** |
|  | Spanning | 1.060 | 0.649 | 1.732 | 0.816 |
| Radiographic Stage | Resectable | (reference) |  |  |  |
|  | Borderline Resectable | 1.819 | 1.078 | 3.066 | **0.025** |
|  | Locally Advanced | 1.819 | 1.098 | 3.015 | **0.020** |
| Initial Neoadjuvant Chemotherapy | FOLFIRINOX | (reference) |  |  |  |
|  | Gemcitabine-Abraxane | 1.235 | 0.802 | 1.901 | 0.338 |
|  | Other Regimen | 0.741 | 0.435 | 1.263 | 0.270 |
| Chemotherapy Switch |  | 1.002 | 0.670 | 1.497 | 0.992 |
| Chemotherapy Side Effects | None | (reference) |  |  |  |
|  | Dose Reduction | 0.857 | 0.608 | 1.209 | 0.380 |
|  | Therapy Stopped | 1.341 | 0.829 | 2.168 | 0.232 |
| Neoadjuvant Radiation |  | 0.762 | 0.538 | 1.079 | 0.125 |
| Attrition | Pancreatectomy | (reference) |  |  |  |
|  | Attrition | 3.023 | 2.106 | 4.339 | **<0.001** |
|  |  |  |  |  |  |
